# mSphere of Influence: Population-level thinking to unravel microbial pathogenicity

**DOI:** 10.1128/msphere.00588-24

**Published:** 2025-08-12

**Authors:** Amelia E. Barber

**Affiliations:** 1 Junior Group Fungal Informatics, Institute of Microbiology, Friedrich Schiller University Jena 9378, Jena, Germany; 2 Cluster of Excellence Balance of the Microverse, Friedrich Schiller University Jena 9378, Jena, Germany; University of Guelph, Guelph, Ontario, Canada

**Keywords:** mycology, microbial pathogenesis, strain heterogeneity

## Abstract

Amelia E. Barber works in the field of fungal genomics and pathogenesis. In this mSphere of Influence article, she reflects on how the paper “Strain heterogeneity in a non-pathogenic *Aspergillus* fungus highlights factors associated with virulence” from the group of Antonis Rokas changed her view on the binary characterization of microbes as pathogens or non-pathogens. The work highlights the overlapping virulence traits shared between the two groups and encourages the use of a population-based framework. To understand the full spectrum of microbial pathogenic potential, virulence phenotypes are characterized across a strain population of isolates from “pathogenic” species and closely related “non-pathogens,” in this case revealing a continuum rather than a clear distinction.

## COMMENTARY

Fungal pathogens pose a serious and growing threat to human health. A striking feature of these organisms is their high interspecies phenotypic heterogeneity, which is underpinned by exceptionally dynamic and plastic genomes. For the WHO priority pathogen *Aspergillus fumigatus*, this includes notable variation between strains in gene content, stress resistance, induction of host responses, and virulence in animal models. Notably, most human fungal pathogens, including *A. fumigatus*, have evolved from non-pathogenic ancestors and therefore remain closely related to non-pathogenic relatives. The study “Strain heterogeneity in a non-pathogenic *Aspergillus* fungus highlights factors associated with virulence” (1) by Antonis Rokas’ group addresses the key research questions of whether these closely related non-pathogen relatives also possess a substantial degree of interspecific heterogeneity, and to what extent does their phenotypic variation overlaps with that of the pathogenic species, particularly under infection-relevant conditions? Strikingly, the authors find that *Aspergillus fischeri*, a close non-pathogenic relative of *A. fumigatus*, displays even greater intraspecific diversity and that this variation frequently overlaps with the pathogenicity-associated phenotypes of *A. fumigatus*. This study challenges the prevailing practice of categorizing the entire species as “pathogens” or “non-pathogens,” which is often based on research conducted using a single reference strain. Instead, it introduces a population-based framework, where the intraspecific diversity of pathogens and closely related species is defined across a diverse set of strains. This approach allows us to learn not only how these organisms cause disease but also how their virulence evolves.

To investigate the degree of strain heterogeneity in the non-pathogen and the boundaries between this and the traits of the pathogen, the study performed extensive genomic, transcriptomic, and phenotypic profiling of 16 “non-pathogenic” *A. fischeri* strains, contextualized through direct comparisons with the pathogen *A. fumigatus*. Strikingly, many *A. fischeri* strains exhibited phenotypes overlapping with those of *A. fumigatus*, including survival within macrophages and induction of inflammatory cytokines. Even more notably, they observed a wide range of virulence in a mouse model of disease. The most lethal *A. fischeri* strains did not quite reach that of the commonly used *A. fumigatus* reference strain A1163 but were likely comparable to Af293, another widely used *A. fumigatus* strain known to be less virulent in animal models. Yet at the genome level, most virulence-associated genes were conserved across *A. fischeri* and *A. fumigatus*, and their presence alone could not predict strain behavior or virulence.

This work challenged the binary framework I had implicitly accepted for thinking about fungal pathogens. By demonstrating substantial overlap in infection-relevant phenotypes between *A. fischeri* and *A. fumigatus*, the study revealed that the pathogenicity is not cleanly constrained by species designations. Rather than discrete categories, “pathogenic” and “non-pathogenic” strains exist along a continuum that is shaped by their aggregated genomic and phenotypic profile. This has inspired me to adopt a population-level model for my own research in microbial virulence, emphasizing the characterization of multiple strains within and across species to map the phenotypic landscape and pathogenic potential. The study raises critical questions about how many other “non-pathogenic” fungal species harbor strains with latent or underappreciated virulence traits and whether this contributes to clinical outcomes in ways that are currently overlooked. In clinical settings, samples are often not accurately identified to the species level due to time and cost constraints. This work also suggests that organisms currently classified as non-pathogens may already possess a broad repertoire of virulence-associated traits and may not require substantial change to become problematic infectious diseases. In summary, this work encourages microbial pathogen researchers to adopt a population-level framework by underscoring the limitations of generalizing from single reference strains and stopping our analyses at species boundaries. I look forward to using this exciting paradigm to study microbial virulence and how it evolves in my future research.
